# Empirical sample-specific approaches to define HPV16 and HPV18 seropositivity in unvaccinated, young, sexually active women

**DOI:** 10.1128/spectrum.00229-24

**Published:** 2024-04-30

**Authors:** Kristy Ng, Samantha Morais, Michel D. Wissing, Ann N. Burchell, Pierre-Paul Tellier, François Coutlée, Tim Waterboer, Mariam El-Zein, Eduardo L. Franco

**Affiliations:** 1 Division of Cancer Epidemiology, McGill University, Montreal, Quebec, Canada; 2 Department of Family and Community Medicine and MAP Centre for Urban Health Solutions, Li Ka Shing Knowledge Institute, St. Michael’s Hospital, Unity Health Toronto, Toronto, Ontario, Canada; 3 Department of Family and Community Medicine, Faculty of Medicine, University of Toronto, Toronto, Ontario, Canada; 4 Department of Family Medicine, McGill University, Montreal, Quebec, Canada; 5 Laboratoire de Virologie Moléculaire, Centre de recherche du Centre Hospitalier de l'Université de Montréal, Montreal, Quebec, Canada; 6 Départements de Microbiologie, Infectiologie et Immunologie, et de Gynécologie‐Obstétrique, Université de Montréal, Montreal, Quebec, Canada; 7 Départements de Médecine, de Médecine clinique de Laboratoire et d'Obstétrique‐Gynécologie, Centre Hospitalier de l'Université de Montréal, Montreal, Quebec, Canada; 8 Infections and Cancer Epidemiology Division, German Cancer Research Center, Heidelberg, Germany; University of Miami, Miami, Florida, USA

**Keywords:** human papillomavirus, humoral immunity, serology, antibodies, mixture model

## Abstract

Given low seroconversion rates following human papillomavirus (HPV) infection, fixed external cutoffs may lead to errors in estimating HPV seroprevalence. We evaluated finite mixture modeling (FMM) and group-based trajectory modeling (GBTM) among unvaccinated, sexually active, HPV-exposed women to determine study-specific HPV16 and HPV18 seropositivity thresholds. We included 399 women (aged 18–24 years) enrolled in the HPV Infection and Transmission Among Couples Through Heterosexual Activity (HITCH) cohort study between 2005 and 2011 in Montreal, Canada. Participants’ blood samples from up to six visits spanning 2 years were tested by multiplex serology for antibodies [median fluorescence intensity (MFI)] specific to bacterially expressed HPV16 and HPV18 L1 glutathione S-transferase fusion proteins. We applied FMM and GBTM to baseline and longitudinal antibody titer measurements, respectively, to define HPV type-specific seronegative and seropositive distributions. Study-specific thresholds were generated as five standard deviations above the mean seronegative antibody titers, mimicking cutoffs (HPV16: 422 MFI; HPV18: 394 MFI) derived from an external population of sexually inactive, HPV DNA-negative Korean women (aged 15–29 years). Agreement (kappa) of study-specific thresholds was evaluated against external cutoffs. Seroprevalence estimates using FMM (HPV16: 27.5%–43.2%; HPV18: 21.7%–49.5%) and GBTM (HPV16: 11.8%–11.8%; HPV18: 9.9%–13.4%) thresholds exceeded those of external cutoffs (HPV: 10.2%; HPV18: 9.7%). FMM thresholds showed slight-to-moderate agreement with external cutoffs (HPV16: 0.26%–0.46%; HPV18: 0.20%–0.56%), while GBTM thresholds exhibited high agreement (HPV16: 0.92%–0.92%; HPV18: 0.82%–0.99%). Kappa values suggest that GBTM, used for longitudinal serological data, and otherwise FMM, for cross-sectional data, are robust methods for determining the HPV serostatus without prior classification rules.

**IMPORTANCE:**

While human papillomavirus (HPV) seropositivity has been employed as an epidemiologic determinant of the natural history of genital HPV infections, only a fraction of women incidentally infected with HPV respond by developing significant antibody levels. HPV seropositivity is often determined by a dichotomous fixed cutoff based on the seroreactivity of an external population of women presumed as seronegative, given the lack of evidence of HPV exposure. However, considering the variable nature of seroreactivity upon HPV infection, which arguably varies across populations, such externally defined cutoffs may lack specificity to the population of interest, causing inappropriate assessment of HPV seroprevalence and related epidemiologic uses of that information. This study demonstrates that finite mixture modeling (FMM) and group-based trajectory modeling (GBTM) can be used to independently estimate seroprevalence or serve as the basis for defining study-specific seropositivity thresholds without requiring prior subjective assumptions, consequently providing a more apt internally valid discrimination of seropositive from seronegative individuals.

## INTRODUCTION

The humoral immune response is likely to play a critical role in the prevention and clearance of human papillomavirus (HPV) infections (1), the most common sexually transmitted infection worldwide (2). Previous studies have shown that HPV vaccination efficacy among non-exposed individuals is largely due to elicited antibody-mediated responses (3, 4). Likewise, persistent natural HPV infections can induce systemic humoral responses that often target the L1 capsid protein and can persist by immunological memory even after the infection has cleared (5, 6). As a result, HPV type-specific seropositivity has been used as an epidemiologic determinant of the natural history of HPV infections and for the evaluation of HPV vaccine efficacy (4, 7). While persistent HPV infection is associated with seroconversion, women incidentally infected with HPV seem to display seroconversion rates of approximately 50% for high-risk types (5, 8–10), suggesting a heterogeneous nature to seroreactivity upon HPV exposure.

HPV serology studies often dichotomize continuous antibody titer data (or equivalent measures of seroreactivity) to estimate the seroprevalence by using a fixed cutoff that is multiple standard deviations (SDs) above the geometric mean antibody titer of an assumed seronegative population, protecting test specificity (6, 8). However, fixed external cutoffs for infections that elicit weak or variable serological responses, like HPV, are not specific to the population of interest. Therefore, their application may lead to inappropriate assessment of HPV seroprevalence and related epidemiologic uses of that information (11). The use of sample-specific, data-driven approaches to distinguish heterogeneity in seroreactivity may be more appropriate for setting a seropositivity threshold for continuous HPV serological data.

As proof of concept, we aimed to compare seroprevalence estimates determined by study-specific seropositivity thresholds with externally defined reference cutoffs. The latter were derived from antibody titers of a group of young women aged 15–29 years from South Korea who reported never engaging in penetrative sexual intercourse nor had any evidence of vaginal HPV DNA for the tested HPV types (8). The study-specific seropositivity thresholds for HPV16 and HPV18, the two most common high-risk HPV genotypes among precancerous cervical lesions (12), were based on data from unvaccinated, sexually active women in the HPV Infection and Transmission Among Couples Through Heterosexual Activity (HITCH) cohort, which was established to investigate HPV transmissibility among young, recently formed couples (13). Specifically, we developed dichotomous seropositivity thresholds using two-component finite mixture modeling (FMM) and group-based trajectory modeling (GBTM), both of which have become increasingly popular in clinical and epidemiological research (14–18). These models suggest probabilistic groupings of individuals who share statistically distinctive characteristics without requiring *a priori* assumptions or the use of subjective assignment rules (19). We also assessed the agreement and performance of the study-specific thresholds relative to the external reference cutoffs for defining the serostatus.

## MATERIALS AND METHODS

### Study population

We used data from women who participated in the HITCH cohort study, the details of which were described previously (13). Briefly, we enrolled 502 heterosexual couples (an assigned male at birth with an assigned female at birth) between 2005 and 2011 in Montreal, Canada. Couples were eligible if the woman was a university or junior college student aged 18–24 years, had started a sexual relationship with a male partner within 6 months prior to enrollment, was neither pregnant nor planning to become pregnant in the following 2 years, had an intact uterus, and had no history of cervical lesions or cancer. Women attended up to six study visits over the 2-year follow-up period (i.e., 0, 4, 8, 12, 18, and 24 months), where they provided nurse-collected blood samples for HPV antibody testing. In addition, female participants completed a total of 11 self-administered web-based questionnaires during the follow-up period (i.e., one every 2 months in the first year and one every 3 months in the second year), which captured repeated measurements of demographics, sexual behavior and history, and HPV vaccination. In the current analysis, we only included female participants reported as unvaccinated at baseline and throughout follow-up and with at least one collected blood sample (*n* = 399).

The HITCH study abides by national and international guidelines regarding research with human data and materials, including the Declaration of Helsinki. The study was conducted in accordance with the principles and articles specified by the Tri-Council Policy Statement Ethical Conduct for Research Involving Humans.

### HPV multiplex serology

Sera were tested for reactivity of antibodies specific to the major capsid protein (L1; HPV16 and HPV18) using a glutathione S-transferase (GST) fusion protein-based multiplex serology assay at the German Cancer Research Center (DKFZ) in Heidelberg, Germany (20). In brief, HPV16 and HPV18 L1 proteins were expressed in *Escherichia coli* as GST-L1-tag fusion proteins and loaded onto sets of glutathione-derivatized, spectrally distinct polystyrene beads (Luminex). Sera were preincubated with polyvinyl alcohol, polyvinylpyrrolidone, and Super ChemiBlock (Chemicon) to block nonspecific binding of antibodies to beads and then incubated with the bead sets. Bound antibodies were detected with a triple-specific biotinylated anti-human immunoglobulin A (IgA), IgM and IgG secondary antibodies, and streptavidin–R-phycoerythrin. Primary serum antibodies were quantified with a Luminex 200 flow cytometer that determines the median fluorescence intensity (MFI). Hereafter, HPV16 and HPV18 GST-L1 specific antibodies will be referred to as HPV16 and HPV18 antibodies, respectively.

### Statistical analyses

Descriptive statistics (mean ± SD or number and percentage) were used to summarize women’s characteristics at the baseline. All analyses were carried out separately for HPV16 and HPV18 antibody titers. We generated, by visit number, histograms on a logarithmic scale to visualize the skewed distribution of serum antibodies (MFI). We also plotted participants’ log-transformed antibody titer trajectories for visual inspection of seroreactivity tendencies over time. Modeling was carried out using log-transformed values, and FMMs and GBTMs were used to define seropositivity thresholds. Both models have been described at length elsewhere (16, 19, 21). The FMM identifies distinctive clusters of individuals by applying a mixture of single-group models within the frequency distribution of HPV antibody titers at the baseline. The GBTM is an application of FMMs that clusters individuals who share similar trajectories of HPV antibody titers over time.

We fitted FMMs according to the procedure from previous studies in order to estimate the results of expectation maximization for combinations of scale mixture of skew-normal distributions, which provide increased flexibility to adapt to antibody titer distributions that are typically skewed (16, 21). We limited the model to categorize the baseline serology data (*n* = 382) into two groups to represent those that are seronegative and seropositive and assumed they both followed normal, skew-normal, or skew-T distributions. We visually inspected the fit of FMMs against the antibody titer distributions at baseline. Following Nagin’s procedure for GBTM selection (19), we estimated group antibody titer trajectories for the longitudinal data (*n* = 399) using the censored normal distribution with censors set at antibody titer values beyond the range of the data (minimum log-transformed value = 0; maximum log-transformed value = 10). GBTM assumes any missing data are unrelated to the outcome and fits the model using maximum likelihood estimation (15). We also limited the model to include two group trajectories and tested zero-order, linear, quadratic, and cubic specifications for the trajectory shapes. We maintained model adequacy by ensuring average posterior probability values > 0.7 for each group (19), subgroups contained >5% of individuals (18), and assessed the GBTM’s ability to distinguish serostatus parsimoniously by visualizing group trajectories dependent on the probability of group membership and individual participant trajectories grouped by maximum posterior probability assignment. Model fit for both FMMs and GBTMs was statistically evaluated using the Akaike information criterion (AIC) and Bayesian information criterion (BIC), calculated so that a smaller value is indicative of a better model (22, 23).

Once model parameters were estimated, we calculated the mean and SD of the seronegative group at baseline (i.e., the probabilistic grouping of women with the lowest antibody titer in each model). We mimicked the methods for developing fixed cutoffs by calculating study-specific seropositivity thresholds for HPV16 and HPV18 that were two, three, four, and five SDs above the mean. Using the baseline HPV antibody titers, we 1) determined HPV seroprevalence among HITCH unvaccinated women based on each study-specific threshold; 2) calculated kappa-statistic measures of agreement and corresponding 95% confidence intervals (CI) to estimate the concordance between the study-specific thresholds and external reference cutoffs (24); and 3) compared the performance of the seropositivity thresholds against the external reference cutoffs using the Youden index (25). The external reference cutoffs (HPV16: 422 MFI; HPV18: 394 MFI) were five SDs above the mean seronegative MFI (8).

Statistical analyses were performed in Stata 18.0 (StataCorp LLC, College Station, Texas); the traj plugin was used for GBTMs (26). Histograms and FMMs were generated in R statistical software (27), the latter using the mixsmsn package (21).

## RESULTS

Characteristics of the 399 participants at baseline are shown in Table S1. The mean age was 21 years (SD = 1.8 years). Most individuals were White (81.4%), and 61.9% had never smoked. The median ages at menarche and first sexual intercourse were 13 years (interquartile range, IQR = 12–13 years) and 17 years (IQR = 15–18 years), respectively. The median lifetime number of sexual partners (partners of oral, vaginal, and/or anal sex) was 6 (IQR = 3–10), and 89.4% of participants had never been pregnant.

**Fig 1:**
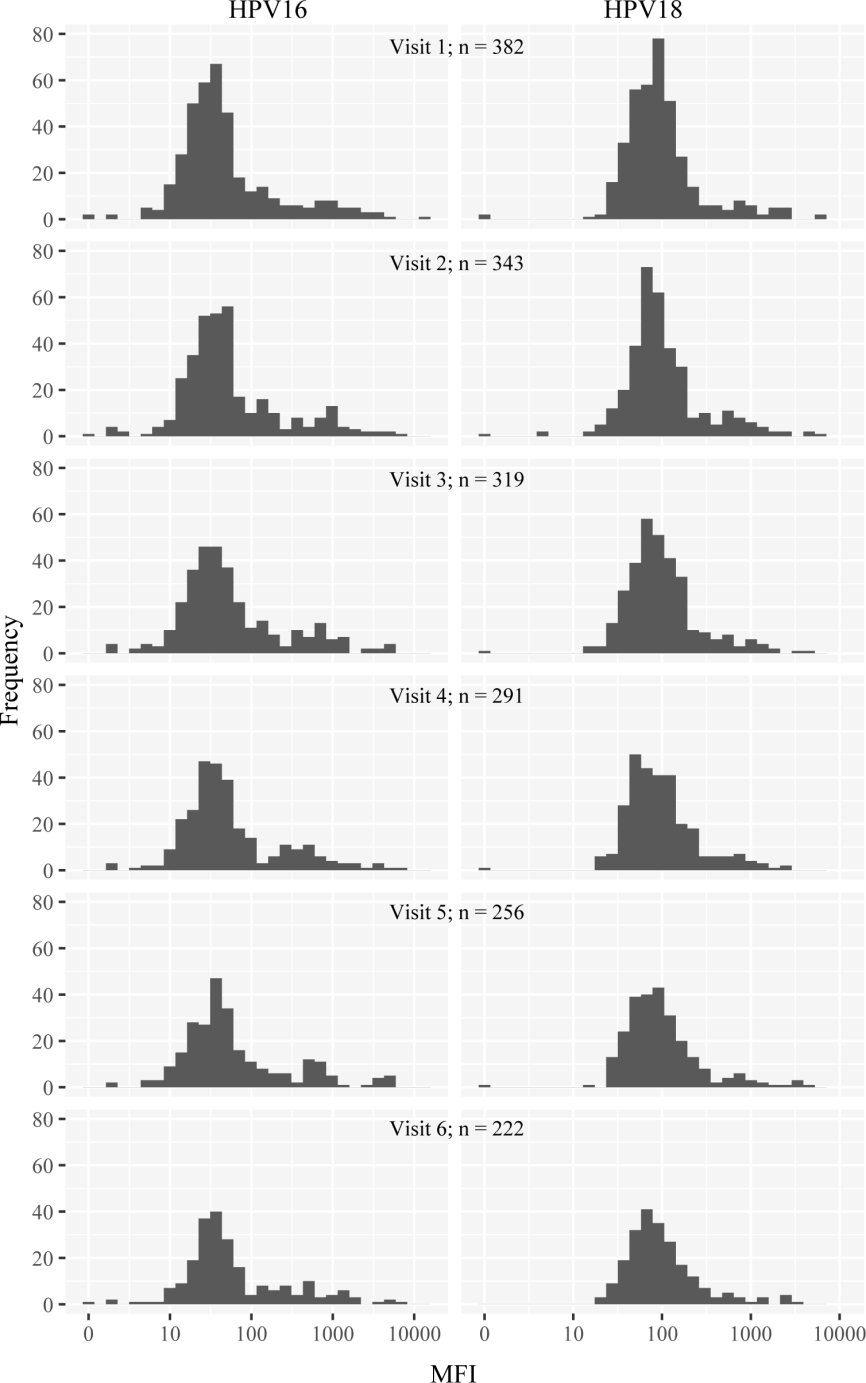
HPV16 and HPV18 antibody titers among unvaccinated women in the HITCH cohort study. The histograms show the frequency distribution of participants (y-axis) by antibody seroreactivity at each study visit measured with HPV multiplex serology for HPV16 and HPV18 and expressed as the median fluorescence intensity (x-axis). Serology data were available for 382 women at baseline. Visit numbers: 1: baseline; 2: 4 months; 3: 8 months; 4: 12 months; 5: 18 months; 6: 24 months. HPV, human papillomavirus; MFI, median fluorescence intensity.

**Fig 2:**
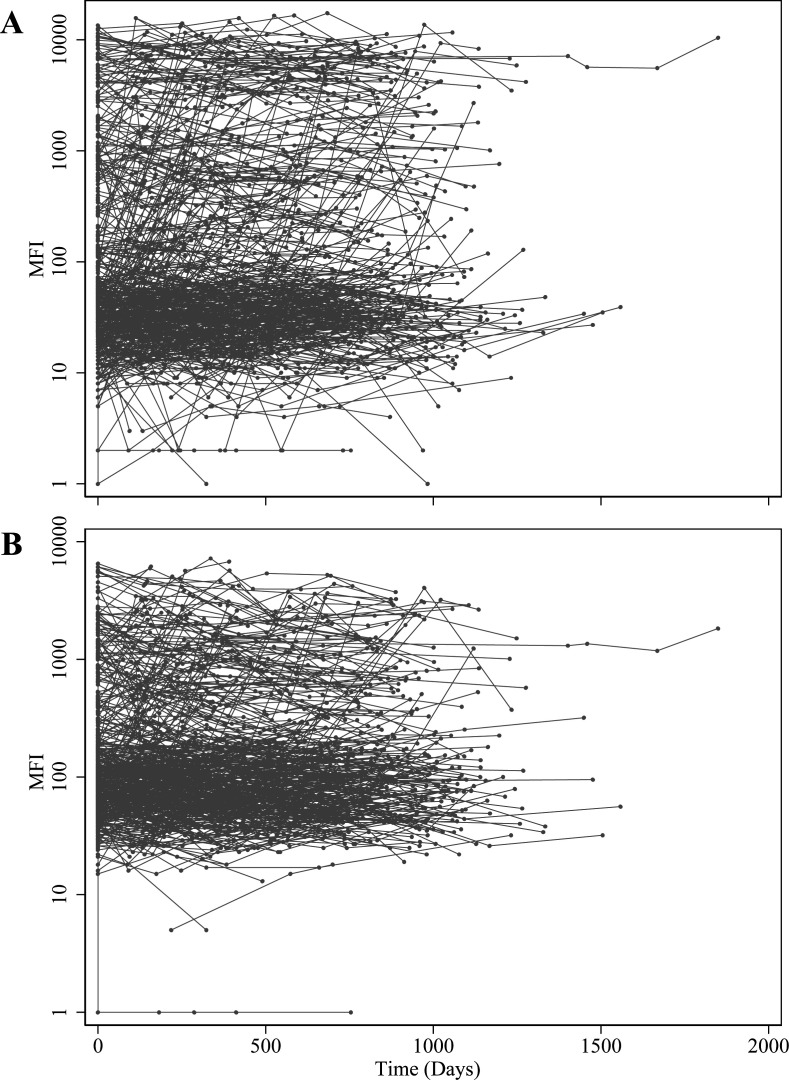
Participants’ trajectories (*n* = 399) of HPV16 and HPV18 antibody titers over follow-up time in the HITCH cohort study. The trajectory plots show the antibody seroreactivity of each unvaccinated woman, measured by HPV multiplex serology for HPV16 (panel A) and HPV18 (panel B) and expressed as the median fluorescence intensity (y-axis) over follow-up time in days (x-axis). HPV, human papillomavirus; MFI, median fluorescence intensity.

Histograms of HPV16 and HPV18 antibody titers at each study visit are presented in Fig. 1, for which 382 (95.7%) of participants provided serological data at baseline. Overall, HPV16 and HPV18 antibody titer distributions were positively skewed, with that of HPV16 showing slight bimodality. The distributions did not exhibit consistent changes in the pattern over time. Participants’ levels of HPV16 and HPV18 antibody titers over follow-up also suggested positive skewness, with trajectories of higher seroreactivity distanced from the cluster of trajectories exhibiting low seroreactivity, presumed to be seronegative individuals (Fig. 2). Visually, trajectories showed no clear change in antibody titers over time.

When modeling cross-sectional baseline serology data with FMMs, the model with the lowest AIC and BIC for HPV16 seroreactivity was the skew-normal model (Table S2). This model categorized the largest percentage of participants as seropositive for HPV16 (Fig. S1). For HPV18, the skew-T model had the lowest AIC and BIC and likewise categorized the largest percentage of participants as seropositive for HPV18. Given the stable nature of participants’ trajectories when considering longitudinal data, the zero-order GBTM for both HPV16 and HPV18 antibody titers showed the lowest BIC score, which increased along with the order of the trajectory shapes (Table S3). The same trend was observed in AIC for HPV18 antibody titers, while the quadratic model had the lowest AIC for HPV16 antibody titers. Despite slight variations in GBTM fit indices for HPV16 group trajectories, the linear, quadratic, and cubic models resulted in identical groupings of seronegative and seropositive participants (Fig. S2). According to the estimated probability of group membership, 81.3% of the study population followed the seronegative trajectory, and the maximum posterior probabilities assigned 325 women to the seronegative group. The zero-order model generated similar groupings, with an estimated seronegative group comprising 81.6% of the study population and a grouping of 327 women based on maximum posterior probabilities. For HPV18, probabilities of group membership were consistent across zero-order, linear, and quadratic GBTMs, generating identical seronegative and seropositive groups. The HPV18 cubic GBTM estimated that 63.0% of the study population followed the seronegative trajectory, with 270 women being assigned to this group based on maximum posterior probabilities.

Table 1 provides the mean and SD of baseline HPV16 and HPV18 antibody titers for participants assigned to the seronegative group along with corresponding study-specific seropositivity thresholds. Since FMMs of differing distributions did not categorize identical seronegative groups for either HPV16 or HPV18, there was variation in FMM-derived means and study-specific thresholds. FMMs with the best statistical fit had seronegative means that were comparable to those of the GBTMs for HPV16 and HPV18. The mean HPV16 antibody titer, based on the zero-order GBTM, was 40.7 MFI (SD = 52.8) and based on either the linear, quadratic, or cubic distribution was 40.9 MFI (SD = 53.0). For HPV18, the zero-order, linear, and quadratic GBTMs also had consistent means (88.0 MFI, SD = 51.3), while that for the cubic distribution was 69.2 MFI (SD = 30.7). External reference cutoffs (HPV16: 422 MFI; HPV18: 394 MFI) were most similar to GBTM thresholds derived from the best fitted models and with the same added multiple of five SDs as the external reference cutoffs (HPV16: 305.9 MFI; HPV18: 344.5 MFI).

**TABLE 1:** Study-specific thresholds for HPV16 and HPV18 seropositivity, based on the mean and standard deviation of unvaccinated seronegative women at baseline in the HITCH cohort study, as estimated via two models and different distribution assumptions^*a*^

HPV type	Model^*b*^	Distribution	Mean (MFI)	SD (MFI)	Study-specific thresholds (MFI)^*c*^			
					+2SDs	+3SDs	+4SDs	+5SDs
HPV16	Finite mixture	Normal	29.9	2.2	34.3	36.5	38.7	40.9
		Skew-normal	47.6	2.2	52.0	54.2	56.4	58.6
		Skew-T	34.6	1.8	38.2	40.0	41.8	43.6
	Group-based trajectory	Zero-order	40.7	52.8	146.3	199.1	251.9	304.7
		Linear	40.9	53.0	146.9	199.9	252.9	305.9
		Quadratic	40.9	53.0	146.9	199.9	252.9	305.9
		Cubic	40.9	53.0	146.9	199.9	252.9	305.9
HPV18	Finite mixture	Normal	77.1	2.0	81.1	83.1	85.1	87.1
		Skew-normal	137.7	2.4	142.5	144.9	147.3	149.7
		Skew-T	85.7	1.7	89.1	90.8	92.5	94.2
	Group-based trajectory	Zero-order	88.0	51.3	190.6	241.9	293.2	344.5
		Linear	88.0	51.3	190.6	241.9	293.2	344.5
		Quadratic	88.0	51.3	190.6	241.9	293.2	344.5
		Cubic	69.2	30.7	130.6	161.3	192.0	222.7

^*a*^ HPV, human papillomavirus; MFI, median fluorescence intensity; SDs, standard deviations.

^*b*^ Serological data of unvaccinated women at baseline (n = 382) and over a 2-year follow-up (n=399) were fitted with finite mixture and group-based trajectory models, respectively, to identify two groups. The seronegative group was identified as the grouping of women with the lowest antibody titer in each model.

^*c*^ Study-specific thresholds were calculated as two, three, four, and five SDs above the mean antibody titer of the seronegative group at baseline (i.e., threshold = mean + (X SD), X = 2, 3, 4, or 5).

As shown in Table 2, increasing the study-specific threshold value (two, three, four, and five SDs above the mean) led to a decrease in baseline seroprevalence for HPV16 and HPV18. FMM-generated thresholds yielded higher seroprevalence estimates relative to GBTM thresholds of the same added multiple of SDs, largely due to the FMM’s smaller SDs. While the zero-order GBTM HPV16 seronegative group had a slightly lower mean than those of the other GBTM distributions, all GBTMs HPV16 thresholds led to identical seroprevalence results. All baseline seroprevalence estimates based on study-specific thresholds exceeded those from external reference cutoffs (Table S4). The highest GBTM thresholds (+ five SDs) gave the lowest seroprevalence estimates of 11.8% (95% CI, 8.7–15.4%) and 9.9% (95% CI, 7.1–13.4%) for HPV16 (zero-order, linear, quadratic, and cubic) and HPV18 (zero-order, linear, and quadratic) at baseline, respectively, which were mostly similar to the seroprevalence estimates generated by the external reference cutoffs (corresponding values of 10.2% and 9.7%).

**TABLE 2:** HPV16 and HPV18 seroprevalence at baseline among unvaccinated women in the HITCH cohort study by the study-specific threshold, as estimated via two models and various data distributions^*a*^

HPV type	Model	Distribution	Seroprevalence by the study-specific threshold^*b*^ (%, 95% CI)			
			+2SDs	+3SDs	+4SDs	+5SDs
HPV16	Finite mixture	Normal	50.0 (44.9–55.1)	46.6 (41.5–51.7)	44.2 (39.2–49.4)	43.2 (38.1–48.3)
		Skew-normal	33.3 (28.5–38.2)	31.2 (26.5–36.1)	30.1 (25.5–35.0)	27.5 (23.1–32.3)
		Skew-T	44.2 (39.2–49.4)	43.2 (38.2–48.3)	42.4 (37.4–47.5)	39.3 (34.3–44.4)
	Group-based trajectory	Zero-order	16.2 (12.7–20.3)	14.1 (10.8–18.0)	13.4 (10.1–17.2)	11.8 (8.7–15.4)
		Linear	16.2 (12.7–20.3)	14.1 (10.8–18.0)	13.4 (10.1–17.2)	11.8 (8.7–15.4)
		Quadratic	16.2 (12.7–20.3)	14.1 (10.8–18.0)	13.4 (10.1–17.2)	11.8 (8.7–15.4)
		Cubic	16.2 (12.7–20.3)	14.1 (10.8–18.0)	13.4 (10.1–17.2)	11.8 (8.7–15.4)
HPV18	Finite mixture	Normal	53.1 (48.0–58.2)	52.4 (47.2–57.5)	51.1 (45.9–56.2)	49.5 (44.4–54.6)
		Skew-normal	22.3 (18.2–26.8)	22.0 (17.9–26.5)	22.0 (17.9–26.5)	21.7 (17.7–26.2)
		Skew-T	46.6 (41.5–51.7)	46.3 (41.3–51.5)	44.2 (39.2–49.4)	42.7 (37.7–47.8)
	Group-based trajectory	Zero-order	15.7 (12.2–19.8)	12.3 (9.2–16.0)	10.7 (7.8–14.3)	9.9 (7.1–13.4)
		Linear	15.7 (12.2–19.8)	12.3 (9.2–16.0)	10.7 (7.8–14.3)	9.9 (7.1–13.4)
		Quadratic	15.7 (12.2–19.8)	12.3 (9.2–16.0)	10.7 (7.8–14.3)	9.9 (7.1–13.4)
		Cubic	25.9 (21.6–30.6)	19.9 (16.0–24.3)	15.2 (11.7–19.2)	13.4 (10.1–17.2)

^*a*^ HPV, human papillomavirus; MFI, median fluorescence intensity; SDs, standard deviations.

^*b*^ Serological data of unvaccinated women at baseline (n = 382) and over a 2-year follow-up (n = 399) were fitted with finite mixture and group-based trajectory models, respectively, to identify two groups. The seronegative group was identified as the grouping of women with the lowest antibody titer in each model. Study-specific thresholds were calculated as two, three, four, and five SDs above the mean antibody titer of the seronegative group at baseline, as shown in Table 1.

As expected, an agreement between the study-specific thresholds and external reference cutoffs was weakest with the lowest thresholds (+ two SDs) and increased with increase in thresholds (Table 3). Kappa values of FMM thresholds were generally lower than those of GBTM thresholds. All GBTM thresholds of the lowest values (+ two SDs) were in substantial agreement (kappa between 0.61 and 0.80) with the external reference cutoffs, apart from the HPV18 cubic GBTM threshold. Agreement increased to almost perfect (kappa >0.81), with additional SDs added to the mean for all GBTM thresholds.

**TABLE 3:** Agreement^*a*^ between study-specific thresholds^*b*^ and external reference cutoffs^*c*^ for HPV16 and HPV18 seropositivity among unvaccinated women in the HITCH cohort study at baseline (*n* = 382)^*d*^

HPV type	Model	Distribution	Kappa (95% CI)			
			+2SDs	+3SDs	+4SDs	+5SDs
HPV16	Finite mixture	Normal	0.20 (0.15–0.26)	0.23 (0.17–0.30)	0.25 (0.18–0.32)	0.26 (0.19–0.33)
		Skew-normal	0.37 (0.28–0.46)	0.40 (0.31–0.50)	0.42 (0.32–0.51)	0.46 (0.36–0.56)
		Skew-T	0.25 (0.18–0.32)	0.26 (0.19–0.33)	0.27 (0.20–0.34)	0.30 (0.22–0.38)
	Group-based trajectory	Zero-order	0.74 (0.64–0.84)	0.82 (0.73–0.91)	0.85 (0.77–0.93)	0.92 (0.86–0.98)
		Linear	0.74 (0.64–0.84)	0.82 (0.73–0.91)	0.85 (0.77–0.93)	0.92 (0.86–0.98)
		Quadratic	0.74 (0.64–0.84)	0.82 (0.73–0.91)	0.85 (0.77–0.93)	0.92 (0.86–0.98)
		Cubic	0.74 (0.64–0.84)	0.82 (0.73–0.91)	0.85 (0.77–0.93)	0.92 (0.86–0.98)
HPV18	Finite mixture	Normal	0.17 (0.12–0.23)	0.18 (0.12–0.23)	0.19 (0.13–0.24)	0.20 (0.14–0.26)
		Skew-normal	0.55 (0.44–0.65)	0.55 (0.44–0.66)	0.55 (0.44–0.66)	0.56 (0.45–0.67)
		Skew-T	0.22 (0.16–0.28)	0.22 (0.16–0.29)	0.24 (0.17–0.31)	0.25 (0.18–0.32)
	Group-based trajectory	Zero-order	0.73 (0.63–0.83)	0.87 (0.79–0.95)	0.94 (0.89–1.00)	0.99 (0.96–1.00)
		Linear	0.73 (0.63–0.83)	0.87 (0.79–0.95)	0.94 (0.89–1.00)	0.99 (0.96–1.00)
		Quadratic	0.73 (0.63–0.83)	0.87 (0.79–0.95)	0.94 (0.89–1.00)	0.99 (0.96–1.00)
		Cubic	0.47 (0.37–0.57)	0.60 (0.50–0.71)	0.75 (0.65–0.85)	0.82 (0.73–0.91)

^*a*^ Kappa agreement: <0: poor; 0 – 0.20: slight; 0.21 – 0.40: fair; 0.41-–0.60: moderate; 0.61-–0.80: substantial; >0.81: almost perfect.

^*b*^ Serological data of unvaccinated women at baseline (n = 382) and over a 2-year follow-up (n = 399) were fitted with finite mixture and group-based trajectory models, respectively, to identify two groups. The seronegative group was identified as the grouping of women with the lowest antibody titer in each model. Study-specific thresholds were calculated as two, three, four, and five SDs above the mean antibody titer of the seronegative group at baseline, as shown in Table 1.

^*c*^ External reference cut-offs were defined as five SDs above the mean seronegative antibody titer of a group of young women aged 15–29 from South Korea who had reportedly never engaged in penetrative sexual intercourse nor had any evidence of genital HPV DNA for the tested HPV types (8).

^*d*^ CI : confidence interval; HPV : human papillomavirus; SDs : standard deviations.

As shown in the cross-tabulations of seropositivity by study-specific thresholds against external reference cutoffs (Table 4), all seropositivity thresholds were 100% sensitive, correctly identifying all participants considered seropositive according to the external reference cutoffs. As a result, the Youden index was equivalent to the specificity of each study-specific threshold. Youden index values increased with study-specific thresholds, suggesting improvement in performance at the highest threshold (+ five SDs). Overall, FMM thresholds had lower Youden indices than GBTM thresholds. For both HPV16 and HPV18, the +five SD thresholds generated by the skew-normal FMMs correspondingly had the highest specificities of 80.8% (95% CI, 76.8–84.7%) and 86.7% (95% CI, 83.3–90.1%) among all FMM thresholds. The +five SD thresholds generated by GBTMs led to indices of 95.9% or greater.

**TABLE 4:** Cross-tabulations and Youden index (%, 95% CI) of study-specific thresholds^*a*^ with external reference cutoffs^*b*^ that define HPV16 and HPV18 seropositivity among unvaccinated women in the HITCH cohort study at baseline (*n* = 382)^*c*^

HPV type	Model	Distribution	External reference cutoffs	Study-specific thresholds											
				+2SDs			+3SDs			+4SDs			+5SDs		
				Pos.	Neg.	Youden	Pos.	Neg.	Youden	Pos.	Neg.	Youden	Pos.	Neg.	Youden
HPV16	Finite mixture	Normal	Pos.	39	0	55.7 (50.7–60.7)	39	0	59.5 (54.6–64.4)	39	0	62.1 (57.2–67.0)	39	0	63.3 (58.4–68.1)
			Neg.	152	191		139	204		130	213		126	217	
		Skew-normal	Pos.	39	0	74.3 (70.0–78.7)	39	0	76.7 (72.4–80.9)	39	0	77.8 (73.7–82.0)	39	0	80.8 (76.8–84.7)
			Neg.	88	255		80	263		76	267		66	277	
		Skew-T	Pos.	39	0	62.1 (57.2–67.0)	39	0	63.3 (58.4–68.1)	39	0	64.1 (59.3–69.0)	39	0	67.6 (63.0–72.3)
			Neg.	130	213		126	217		123	220		111	232	
	Group-based trajectory	Zero-order	Pos.	39	0	93.3 (90.8–95.8)	39	0	95.6 (93.6–97.7)	39	0	96.5 (94.7–98.3)	39	0	98.3 (96.9–99.6)
			Neg.	23	320		15	328		12	331		6	337	
		Linear	Pos.	39	0	93.3 (90.8–95.8)	39	0	95.6 (93.6–97.7)	39	0	96.5 (94.7–98.3)	39	0	98.3 (96.9–99.6)
			Neg.	23	320		15	328		12	331		6	337	
		Quadratic	Pos.	39	0	93.3 (90.8–95.8)	39	0	95.6 (93.6–97.7)	39	0	96.5 (94.7–98.3)	39	0	98.3 (96.9–99.6)
			Neg.	23	320		15	328		12	331		6	337	
		Cubic	Pos.	39	0	93.3 (90.8–95.8)	39	0	95.6 (93.6–97.7)	39	0	96.5 (94.7–98.3)	39	0	98.3 (96.9–99.6)
			Neg.	23	320		15	328		12	331		6	337	
HPV18	Finite mixture	Normal	Pos.	37	0	51.9 (46.9–55.9)	37	0	52.8 (47.8–57.8)	37	0	54.2 (49.2–59.2)	37	0	55.9 (51.0–60.9)
			Neg.	166	179		163	182		158	187		152	193	
		Skew-normal	Pos.	37	0	86.1 (82.6–89.6)	37	0	86.4 (82.9–89.8)	37	0	86.4 (82.9–89.8)	37	0	86.7 (83.3–90.1)
			Neg.	48	297		47	298		47	298		46	299	
		Skew-T	Pos.	37	0	59.1 (54.2–64.1)	37	0	59.4 (54.5–64.3)	37	0	61.7 (56.9–66.6)	37	0	63.5 (58.7–68.3)
			Neg.	141	204		140	205		132	213		126	219	
	Group-based trajectory	Zero-order	Pos.	37	0	93.3 (90.8–95.8)	37	0	97.1 (95.4–98.8)	37	0	98.8 (97.8–99.9)	37	0	99.7 (99.2–100.3)
			Neg.	23	322		10	335		4	341		1	344	
		Linear	Pos.	37	0	93.3 (90.8–95.8)	37	0	97.1 (95.4–98.8)	37	0	98.8 (97.8–99.9)	37	0	99.7 (99.2–100.3)
			Neg.	23	322		10	335		4	341		1	344	
		Quadratic	Pos.	37	0	93.3 (90.8–95.8)	37	0	97.1 (95.4–98.8)	37	0	98.8 (97.8–99.9)	37	0	99.7 (99.2–100.3)
			Neg.	23	322		10	335		4	341		1	344	
		Cubic	Pos.	37	0	82.0 (78.2–85.9)	37	0	88.7 (85.5–91.9)	37	0	93.9 (91.5–96.3)	37	0	95.9 (94.0–97.9)
			Neg.	62	283		39	306		21	324		14	331	

^*a*^ Serological data of unvaccinated women at baseline (n = 382) and over a 2-year follow-up (n = 399) were fitted with finite mixture and group-based trajectory models, respectively, to identify two groups. The seronegative group was identified as the grouping of women with the lowest antibody titer in each model. Study-specific thresholds were calculated as two, three, four, and five SDs above the mean antibody titer of the seronegative group at baseline, as shown in Table 1.

^*b*^ External reference cutoffs were defined as five SDs above the mean seronegative antibody titer of a group of young women aged 15–29 from South Korea who had reportedly never engaged in penetrative sexual intercourse nor had any evidence of genital HPV DNA for the tested HPV types (8).

^*c*^ CI, confidence interval; HPV, human papillomavirus; neg, negative; pos, positive; SDs, standard deviations.

## DISCUSSION

In this analysis, we explored the use of study-specific thresholds derived from empirical, data-specific modeling on women from the HITCH cohort against fixed seropositivity cutoffs based on an external non-exposed population with *a priori* assumption of seronegativity. Overall, FMMs and GBTMs applied to cross-sectional and longitudinal continuous HPV serological data, respectively, distinguished the likely seronegative individuals in a cohort of unvaccinated women who were sexually active and thus likely exposed to HPV prior to and during the study. Study-specific seropositivity thresholds varied based on the model used, though most were comparable to the fixed external reference cutoffs, particularly those based on longitudinal data. Therefore, our findings provide compelling evidence for the use of GBTM to determine HPV serostatus when longitudinal data are available, and otherwise FMM in the absence of longitudinal data, as ways to avoid the arbitrariness of externally defined cutoffs.

Previous studies reported that HPV exposure may result in seropositivity for only a fraction of individuals, with a modest relationship between seropositivity and HPV DNA positivity (8, 10, 28). For HPV serological data, it is thus assumed that unobserved subgroups for seroreactivity exist regardless of the level of exposure. This assumption is the basis of the FMM, which approximates a continuous distribution and identifies a finite number of distinctive groups without *ad hoc* and *ex ante* classification rules (15, 16, 19). Therefore, such statistical modeling methods for disentangling the seronegative individuals from the rest of the population presents numerous advantages for developing study-specific seropositivity thresholds and estimating the seroprevalence. In fact, the FMM has been effective in estimating the seroprevalence and the prevalence of other test outcomes from continuous cross-sectional data (16, 29–34). Previous studies note that seroprevalence estimates determined by mixture models tend to be higher than those using fixed cutoffs (17, 35, 36). For example, Vink and colleagues reported that HPV16 seroprevalence increased by eight percentage points among women when using a two-component mixture model (20%) as compared with using a fixed cutoff (12%) (17). They suggested that fixed cutoffs may lower seroprevalence estimates and introduce classification errors, particularly given weak serological responses to HPV, as such cutoffs are often set to greater values than the antibody titers of the likely seronegative group to prioritize test specificity over sensitivity (17, 37). Our study finding is consistent with these findings; as study-specific threshold values increased with added SDs and approached external reference cutoff values, seroprevalence decreased, thereby improving agreement and performance.

The humoral immune response to HPV infection is dynamic and dependent on factors such as genotype and whether infections persist (10, 38). Since the HITCH cohort study provides longitudinal serological data, GBTM—a semi-parametric application of the FMM that maps the evolution of an outcome over time (18, 19)—can be used to identify homogeneous groups of seronegative and seropositive individuals based on the similarity of antibody titer trajectories rather than relying simply on cross-sectional data. GBTM has been widely applied to outcomes in psychology and criminology and is emerging in medical research (15, 39–42), but its application to serological responses is novel. Our analysis of HPV16 and HPV18 antibody titer trajectories indicates its stability over the 2 years of follow-up. Previous research also reported relatively stable HPV L1 antibody titers following natural infection in both women and men (10, 43, 44). Most defined antibody levels as a dichotomous outcome, while we followed trajectories on a continuous scale; nevertheless, our findings are in concordance.

To our knowledge, this is the first study that establishes seropositivity thresholds using FMMs and GBTMs for HPV16 and HPV18. Our findings show that the GBTM fit to longitudinal data was more robust than the FMM fit to cross-sectional data, the former often leading to identical or similar groupings of individuals regardless of model parameters and fit. This suggests that longitudinal serological data allow for clearer distinction between subpopulations; serostatus based on at least two measurements is more useful than that based on a single measurement. Following the generation of study-specific thresholds for comparison with external reference cutoffs, we noted that, while baseline seroprevalence estimates from GBTM thresholds were always greater than those of external reference cutoffs, GBTM thresholds had higher overall agreement and performance than FMM thresholds, particularly when comparing all study-specific thresholds five SDs above the seronegative mean. This was partially due to greater SDs of seronegative groups defined by GBTMs. GBTMs not only characterize differences in averages (cluster people based on the closeness of antibody levels) but also in trajectories of the outcome within a population (15, 19), potentially explaining the robustness of model clustering but greater SD in baseline seronegative antibody titers.

Of note, we cannot comment on the true accuracy of FMM and GBTM seropositivity thresholds without a gold standard. Past research on HPV16 and HPV18 seroprevalence in similar populations of young, unvaccinated, sexually active women varied widely; corresponding seroprevalences ranged from 4.9% to 43.0% and from 1.3% to 41.0% across various studies (8, 45, 46). The use of discordant fixed cutoffs likely explains some of this variability. Our study demonstrates how slight variations in defining seropositivity cutoffs can largely impact seroprevalence estimates. Nevertheless, our findings underscore the versatility of mixture modeling, which can either directly estimate study-specific seroprevalence or serve as the foundation for fixed thresholds, adjustable based on the desired specificity and sensitivity through the manipulation of added SDs.

Our study has several limitations that need to be acknowledged. We exclusively considered baseline antibody titers when generating seropositivity thresholds; however, we expect this to have a limited impact on our conclusions since antibody titers were fairly stable over time. In addition, though seroreactivity is a continuous rather than a dichotomous construct, we limited our models to define two groups, seronegative or seropositive, of the same trajectory shape or distribution. This was done to directly compare the results to fixed dichotomous cutoffs. While fixed external cutoffs are more appropriate for individual patient diagnosis, they may lack accuracy in determining population prevalence (17, 35, 36), preventing the assessment of the accuracy of seropositivity thresholds relative to true seroprevalence.

To conclude, this study introduces the use of FMM and GBTM as alternative approaches to external reference cutoffs for HPV seroprevalence estimation, as these mixture modeling methods can discriminate seropositive from seronegative individuals without relying on an external group of non-exposed individuals and resorting to virological outcomes. As a proof of concept, we used FMM and GBTM to identify, for HPV16 and HPV18, the presence of discordant HPV seroreactivity among an unvaccinated and HPV-exposed population of young female adults. We corroborate the application of the FMM for continuous cross-sectional HPV serological data, and in the context of longitudinal data, we propose GBTM as a robust method to distinguish study-specific seronegative and seropositive individuals. These models can independently estimate seroprevalence or serve as the basis for creating study-specific fixed cutoffs.

## Data Availability

HITCH participant consent forms stated that data would be published in aggregate form and individual-level data would only be available to study investigators upon request. To access data, please contact Eduardo Franco at eduardo.franco@mcgill.ca. The protocol for the HITCH cohort study has been published (13).

## SUPPLEMENTAL MATERIAL

Supplemental figures and tables spectrum.00229-24-s0001.docx Fig. S1 and S2; Tables S1-S4.

ASM does not own the copyrights to Supplemental Material that may be linked to, or accessed through, an article. The authors have granted ASM a non-exclusive, world-wide license to publish the Supplemental Material files. Please contact the corresponding author directly for reuse.
