# Supplementary material for: Empirical sample-specific approaches to define HPV16 and HPV18 seropositivity in unvaccinated, young, sexually active women
Source: Microbiol Spectr. 2024 Apr 30;12(6):e00229-24. doi: 10.1128/spectrum.00229-24 (PMC11324019; doi:10.1128/spectrum.00229-24)
Supplement: Supplemental figures and tables — Fig. S1 and S2; Tables S1-S4. [file spectrum.00229-24-s0001.docx]

**Supplementary material**

**Defining HPV16 and HPV18 seropositivity in unvaccinated, young, sexually-active women using empirical sample-specific approaches**

Kristy Ng, Samantha Morais, Michel D Wissing, Ann N Burchell, Pierre-Paul Tellier, François Coutlée, Tim Waterboer, Mariam El-Zein, Eduardo L Franco

**Supplementary Table 1.** Baseline characteristics of unvaccinated women in the HITCH cohort study.

| **Variables** | **Overall**  **(n=399)** |
| --- | --- |
|  |  |
| **Age**, mean (SD) | 21 (1.8) |
| **Ethnicity**, n (%) |  |
| White | 323 (81.4) |
| Asian | 33 (8.3) |
| Latino | 19 (4.8) |
| Black | 12 (3.0) |
| Mixed | 10 (2.5) |
| *Missing* | *2* |
| **Smoking**, n (%) |  |
| Never | 247 (61.9) |
| Ever (current/former) | 152 (38.1) |
| **Age at menarche**, median (IQR) | 13 (12-13) |
| *Missing* | *2* |
| **Age at first sexual intercourse**, median (IQR) | 17 (15-18) |
| *Missing* | *3* |
| **Ever pregnant**, n (%) |  |
| Yes | 42 (10.5) |
| No | 356 (89.4) |
| *Missing* | *1* |
| **Number of lifetime sexual partners^a^**, n (%) |  |
| Median (IQR) | 6 (3-10) |
| 1-5 | 188 (47.2) |
| 6-10 | 110 (27.6) |
| 11-15 | 46 (11.6) |
| ≥16 | 54 (13.6) |
| *Missing* | *1* |

IQR, interquartile range; SD, standard deviation.

^a^ Lifetime sexual partners include the total number of individuals who have engaged in oral, vaginal, and/or anal sex with the study participant.

**Supplementary Table 2.** Model parameter estimates and statistical model fit evaluation of two-component finite mixture models for cross-sectional log-transformed serological data by distribution shape and HPV type (16 and 18) among unvaccinated women in the HITCH cohort study at baseline (n=382).

| **HPV type** | **Distribution** | **Group (j)** | **Parameter estimates** | | | **AIC** | **BIC** |
| --- | --- | --- | --- | --- | --- | --- | --- |
|  |  |  | **Mean (µ_j_)** | **Variance (σ^2^_j_)** | **Shape** |  |  |
| **HPV16** | Normal | 1 | 1.48 | 0.12 | -- | 582.75 | 602.48 |
|  |  | 2 | 2.84 | 0.22 | -- |  |  |
|  | Skew Normal | 1 | 1.68 | 0.11 | -1.95 | 555.86 | 583.48 |
|  |  | 2 | 2.16 | 0.88 | -0.84 |  |  |
|  | Skew T | 1 | 1.54 | 0.07 | -0.35 | 559.11 | 590.67 |
|  |  | 2 | 3.11 | 0.64 | -1.51 |  |  |
| **HPV18** | Normal | 1 | 1.89 | 0.09 | -- | 372.03 | 391.76 |
|  |  | 2 | 3.04 | 0.10 | -- |  |  |
|  | Skew Normal | 1 | 2.14 | 0.15 | -2.02 | 353.89 | 381.51 |
|  |  | 2 | 3.06 | 0.21 | -0.61 |  |  |
|  | Skew T | 1 | 1.93 | 0.06 | -0.30 | 314.08 | 345.64 |
|  |  | 2 | 3.35 | 1.16 | -3.35 |  |  |

AIC, Akaike Information Criterion; BIC, Bayesian Information Criterion; HPV, human papillomavirus.

| **HPV type** | **Normal** | **Skew Normal** | **Skew T** |
| --- | --- | --- | --- |
| **HPV16** | 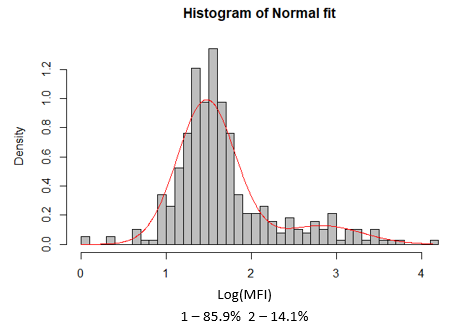 | 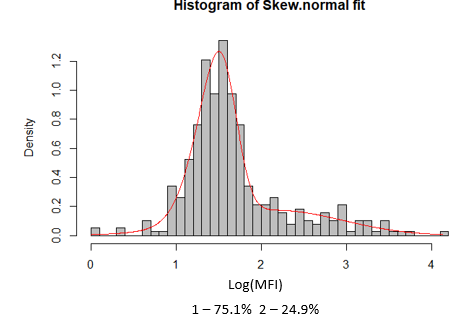 | 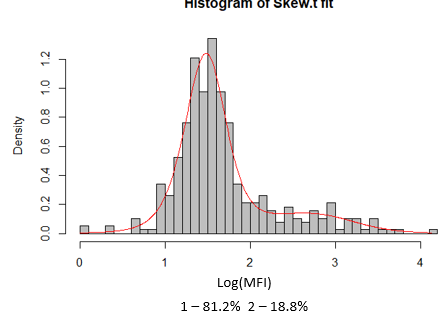 |
| **HPV18** | 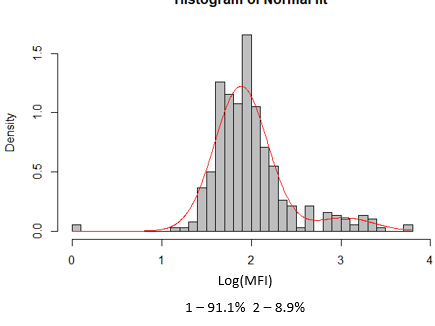 | 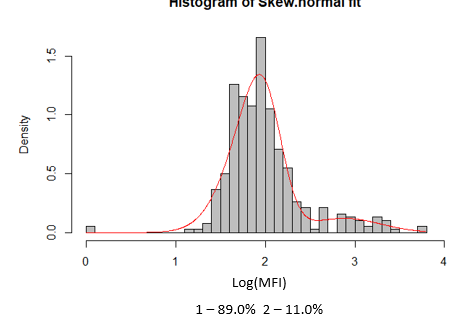 | 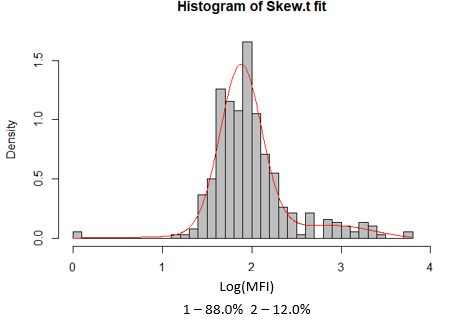 |

**Supplementary Figure 1.** The fit of finite mixture models against the distribution of HPV16 and HPV18 antibody titers among unvaccinated, sexually-active women in the HITCH cohort at baseline (n=382).

**Supplementary Figure 1 legend:**

382 women provided blood samples at the baseline study visit. Cross-sectional serological data at baseline was fitted with two-component finite mixture models of various distribution shapes. The seronegative group was identified as the probabilistic grouping of women with the lowest antibody titer in each model. The histograms show the distribution of antibody titers at baseline measured by HPV multiplex serology for HPV 16 and HPV18 and expressed as median fluorescence intensity (plotted on log_10_ x-axis). The curved line represents the finite mixture model and the percentages in the legend represent the estimated percentage of the study population falling into the seronegative (1) and seropositive (2) group.

HPV, human papillomavirus; MFI, median fluorescence intensity; 1, seronegative group; 2, seropositive group.

**Supplementary Table 3.** Model parameter estimates and statistical model fit evaluation of two-group group-based trajectory models for longitudinal log-transformed serological data by trajectory shape and HPV type (16 and 18) among unvaccinated women in the HITCH cohort study (n=399).

| **HPV type** | **Trajectory shapes** | **Group (j)** | **Parameter estimates**  ${y_{\mathrm{it}}^{*}= \beta}_{0}^{j}+\beta_{1}^{j}\mathrm{Time}_{\mathrm{it}}+\beta_{2}^{j}\mathrm{Time}_{\mathrm{it}}^{2}+\beta_{3}^{j}\mathrm{Time}_{\mathrm{it}}^{3}+ \varepsilon_{\mathrm{it}}$ | | | | **AIC** | **BIC** |
| --- | --- | --- | --- | --- | --- | --- | --- | --- |
|  |  |  | $\beta_{0}^{j}$ | $\beta_{1}^{j}$ | $\beta_{2}^{j}$ | $\beta_{3}^{j}$ |  |  |
| **HPV16** | Zero-order | 1 | 1.50 | -- | -- | -- | 2022.00 | 2037.95 |
|  |  | 2 | 2.77 | -- | -- | -- |  |  |
|  | Linear | 1 | 1.49 | 2.41e-05 | -- | -- | 2015.48 | 2039.41 |
|  |  | 2 | 2.69 | 2.23e-04 | -- | -- |  |  |
|  | Quadratic | 1 | 1.48 | 1.32e-04 | -1.14e-07 | -- | 2013.80 | 2045.72 |
|  |  | 2 | 2.64 | 6.27e-04 | -4.55e-07 | -- |  |  |
|  | Cubic | 1 | 1.48 | 1.77e-04 | -2.27e-07 | 6.77e-11 | 2017.62 | 2057.51 |
|  |  | 2 | 2.64 | 7.45e-04 | -7.89e-07 | 2.27e-10 |  |  |
| **HPV18** | Zero-order | 1 | 1.88 | -- | -- | -- | 958.89 | 974.85 |
|  |  | 2 | 2.84 | -- | -- | -- |  |  |
|  | Linear | 1 | 1.89 | -3.13e-05 | -- | -- | 961.06 | 984.99 |
|  |  | 2 | 2.84 | -1.10e-05 | -- | -- |  |  |
|  | Quadratic | 1 | 1.88 | 3.23e-05 | -6.76e-08 | -- | 962.85 | 994.76 |
|  |  | 2 | 2.86 | -1.99e-04 | 2.08e-07 | -- |  |  |
|  | Cubic | 1 | 1.71 | 2.08e-09 | 4.63e-07 | -3.82e-10 | 1701.35 | 1741.24 |
|  |  | 2 | 2.29 | 2.24e-09 | 6.09e-07 | -5.24e-10 |  |  |

AIC, Akaike Information Criterion; BIC, Bayesian Information Criterion; HPV, human papillomavirus.

Trajectory formula: $y_{\mathrm{it}}^{*}$: individual *i*’s log-transformed antibody titer at time *t*, $\beta_{0}^{j}$: zero-order parameter, $\beta_{1}^{j}$: linear parameter, $\beta_{2}^{j}$: quadratic parameter, $\beta_{3}^{j}$: cubic parameter, j: group, $\varepsilon_{\mathrm{it}}$: disturbance assumed to be normally distributed with a zero mean and constant standard deviation.

|  |  | **GBTM group trajectories** | **GBTM-clustered participant trajectories** | |
| --- | --- | --- | --- | --- |
|  |  |  | **Seronegative** | **Seropositive** |
| **HPV16** | **Zero-order** |  | 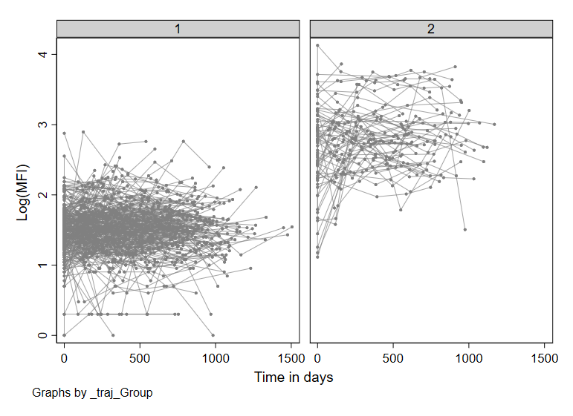 | |
|  | **Linear** |  | 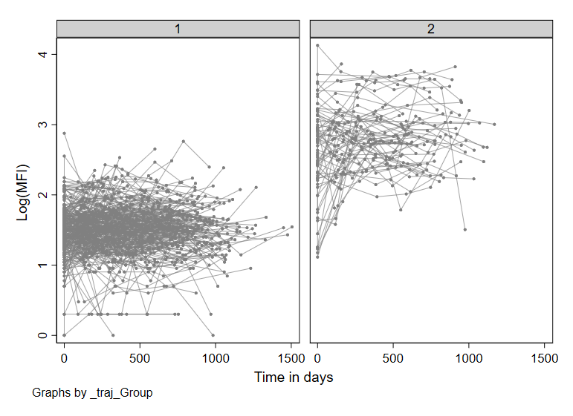 | |
|  | **Quadratic** |  | 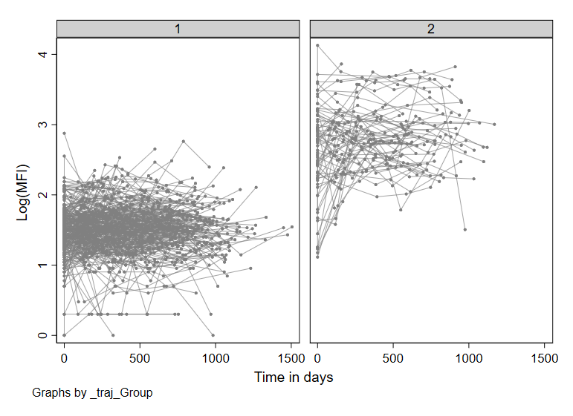 | |
|  | **Cubic** |  | 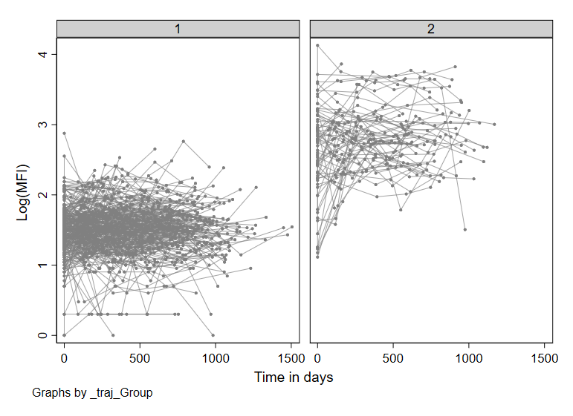 | |
| **HPV18** | **Zero-order** |  | 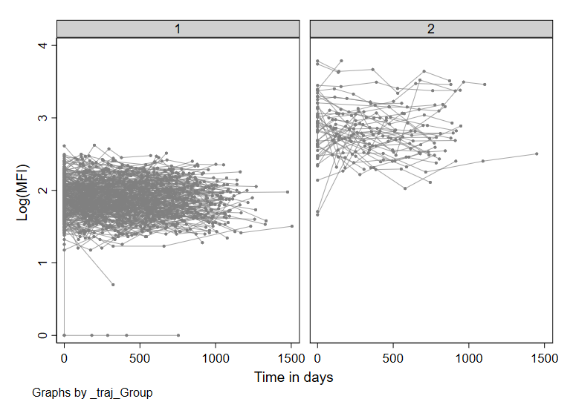 | |
|  | **Linear** |  | 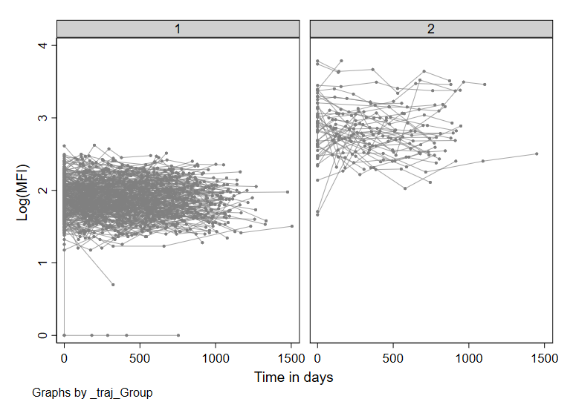 | |
|  | **Quadratic** |  | 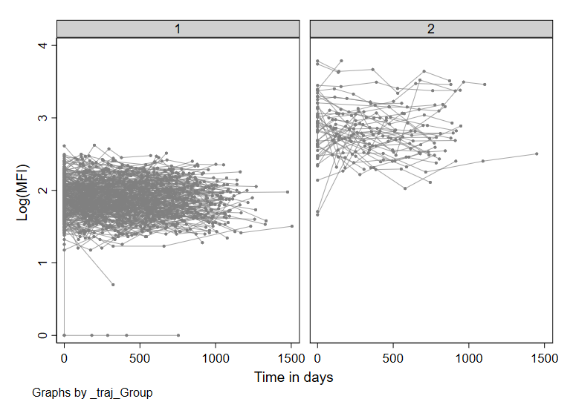 | |
|  | **Cubic** |  | 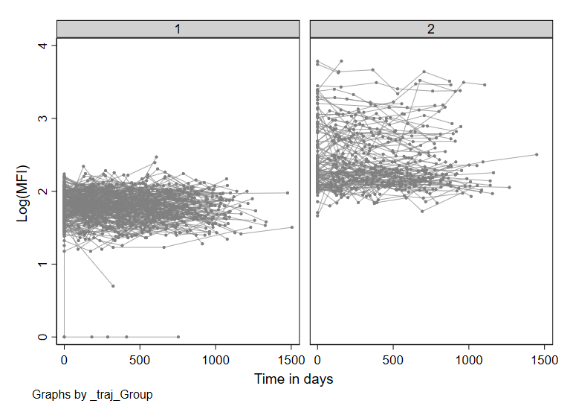 | |

**Supplementary Figure 2.** Group and participant trajectories of seronegative and seropositive groups for HPV16 and HPV18 over time among unvaccinated women in the HITCH cohort study (n=399).

**Supplementary Figure 2 legend:**

399 participants were unvaccinated and provided at least one blood sample over the two-year follow-up. Longitudinal serological data was fitted with GBTMs of various trajectory shapes. The seronegative group was identified as the probabilistic grouping of women with the lowest antibody titer in each model. All trajectory plots display antibody seroreactivity for the seronegative (1) and seropositive (2) groups, measured by HPV multiplex serology for HPV16 and HPV18 and expressed as log_10_ median fluorescence intensity (y-axis) over time in days (x-axis). The group trajectory plots display the mean trajectory for the seronegative and seropositive groups. The grey dotted lines flanking the mean trajectory represent the 95% confidence interval. The proportions in the legend represent the estimated percentage of the study population belonging to each group, determined by the probability of group membership. The clustered participant trajectory plots display the antibody seroreactivity of each individual and categorized into seronegative and seropositive groups, assigned by the maximum posterior probability.

GBTM, group-based trajectory model; HPV, human papillomavirus; 1, seronegative group; 2, seropositive group.

**Supplementary Table 4.** Seroprevalence (n, %) against HPV16 and HPV18 at each visit in unvaccinated, sexually-active women in the HITCH cohort study using external reference cut-offs^a^.

| **Seroprevalence by visit** | | | | | | |
| --- | --- | --- | --- | --- | --- | --- |
| **Visit #** | **1 (n=382)** | **2 (n=343)** | **3 (n=319)** | **4 (n=291)** | **5 (n=256)** | **6 (n=222)** |
| **HPV type** | **n (%)** | **n (%)** | **n (%)** | **n (%)** | **n (%)** | **n (%)** |
| **HPV16** | 39 (10.2) | 39 (11.4) | 41 (12.8) | 34 (11.7) | 39 (15.2) | 30 (13.5) |
| **HPV18** | 37 (9.7) | 40 (11.7) | 28 (8.8) | 28 (9.6) | 22 (8.6) | 19 (8.6) |

HPV, human papillomavirus. Visit numbers: 1: baseline, 2: 4 months, 3: 8 months, 4: 12 months, 5: 18 months, 6: 24 months.

^a^ External reference cut-offs were defined as five standard deviations above the mean seronegative antibody titer of a group of young women aged 15-29 from South Korea who had reportedly never engaged in penetrative sexual intercourse nor had any evidence of genital HPV DNA for the tested HPV types (8).
